# 81 The Burn Team: A Coveted Resource

**DOI:** 10.1093/jbcr/iraf019.081

**Published:** 2025-04-01

**Authors:** Nicole Kopari, Michael Mosier, Francesca Ferrigno

**Affiliations:** UCSF Fresno Leon S. Peters Burn Center; University of California San Francisco Fresno; UCSF Fresno Leon S. Peters Burn Center

## Abstract

**Introduction:**

Surgeon response times are required at most American College of Surgeons verified trauma centers, yet these same standards do not exist for verified burn centers. Although burns are traumatic injuries, not all the same resources are required for even the most severely burned patients. Experiencing frequent over triage, we sought to evaluate the triage of burn patients in our busy emergency department (ED), and then created new notification criteria for burn patients. We present our early experience, which limited over utilization of resources and improved efficiencies of appropriate resources to reflect the severity of injury.

**Methods:**

From April 2024 to August 2024, we tracked the number of burn patients that were paged to the burn team as either a burn activation, response, or consult. Each tiered level of notification required a response time of 15 minutes, 30 minutes, or 60 minutes respectively. Our activation notification required similar resources as a trauma activation including emergency and trauma team members, airway support, operating room staff, radiology, pharmacy, laboratory, and a designated nursing team. If there were concerns for concomitant trauma, these patients were evaluated by the trauma team as a trauma activation.

**Results:**

A total of 199 notifications were paged with only 48% of the patients requiring admission to the burn center. Upon review during the same time period for our trauma patients, roughly 90% of the trauma team pages resulted in admissions. No burn patients required immediate surgical interventions other than escharotomies which were performed both in the ED and upon admission to the burn intensive care unit (BICU). Given the high cost of overutilization of resources, we adjusted the burn notification criteria to limit our burn activations to pre-hospital intubations and burns ≥40% TBSA. The burn response criteria was adjusted to include patients anticipated to require BICU admissions including burns ≥20% TBSA, circumferential full thickness extremity burns, electrical injuries with significant cardiac arrhythmias, and burn patients with concerns for inhalational injury. The remaining burn patients triggered a burn consult. With implementation of the new notification criteria, 100% of the burn activations, 40% of the burn responses, and 43% of the burn consults were admitted.

**Conclusions:**

Changing the notification criteria, has decreased over utilization of limited resources and allowed our burn team to better prepare for admissions to the BICU. Our burn team now readies a BICU bed when an activation is paged, limiting time in the ED for the burn patient and improving efficiencies in care. Further, ongoing educating of our ED colleagues and pre-hospital first responders should allow for further improvements in care.

**Applicability of Research to Practice:**

Adjusting the notification criteria improved the quality of care the critically ill patients received and resulted in an improved resource stewardship.

**Funding for the Study:**

N/A

